# Clinical Swallow Examination Following Laryngectomy: An International e-Delphi Consensus Process

**DOI:** 10.1007/s00455-024-10785-0

**Published:** 2025-01-21

**Authors:** Sarah E. Wilson, Bena Brown, Clare L. Burns

**Affiliations:** 1 Speech Pathology & Audiology Department, Royal Brisbane & Women’s’ Hospital, Level 2, Dr James Mayne Building, Butterfield Street, Herston, Brisbane, QLD Australia; 2 School of Health and Rehabilitation Sciences, The University of Queensland, Brisbane, QLD Australia; 3 Southern Queensland Centre of Excellence in Aboriginal and Torres Strait Islander Primary Health Care, Metro South Health, Brisbane, QLD Australia; 4 School of Public Health, The University of Queensland, Brisbane, QLD Australia

**Keywords:** Laryngectomy, Dysphagia, Deglutition disorders, Clinical swallow examination

## Abstract

Clinical swallow examination (CSE) following laryngectomy (± pharyngeal resection) remains a critical step in dysphagia evaluation. Whilst the core components of a standard CSE service a broad spectrum of patient populations, no evidence exists examining the essential assessment items specific to CSE in the laryngectomy population. The aim of this study was to identify the tasks, measures and observations considered necessary to include in a CSE post laryngectomy. Using an e-Delphi approach, a 4-round online survey series was undertaken with 34 speech pathologists experienced in laryngectomy swallowing management from 6 countries. In the first round (item generation) participants were provided with the questions from the swallowing outcomes after laryngectomy (SOAL) as stimulus, to generate a list of tasks, measures and observations as well as clarifying questions they would ask the patient during a CSE. In the subsequent e-Delphi rounds the participants rated the importance of the compiled assessment items. A total of 34 items were rated of critical importance for inclusion in a laryngectomy CSE by ≥ 75% of participants. Two thirds of the consensus items (23 items) were patient history and interview questions incorporating medical and swallowing history (4 items) and patient interview (19 items). The remaining 11 items related to swallow tasks and observations (9 items) and onward referral (2 items). These 34 consensus items can be considered as a draft framework for laryngectomy CSE to guide clinical practice and research.

**Supplementary Information:**

The online version contains supplementary material available at 10.1007/s00455-024-10785-0.

## Introduction

Laryngectomy surgery involves removal of the larynx (± pharyngeal resection) and permanent separation and redirection of the respiratory and digestive tracts. By changing these structures, the procedure disrupts the natural oropharyngeal pressure systems involved in swallowing. Dysphagia is a well-established and prevalent sequalae post laryngectomy, with more than 70% of patients reporting swallowing difficulties from acute recovery into long-term survivorship [1, 2]. Causes of swallow dysfunction for this population are multifactorial with contributors including the extent of surgical resection, type of closure or reconstruction, surgical complications and previous or post operative radiotherapy [3–5].Radiological and manometric studies of this population have described prolonged bolus transit time and swallow duration as well as persistent neopharyngeal residue secondary to a loss in pressure differentials due to an enlarged neopharyngeal space, reduced negative pressure generation and incomplete relaxation of the UES [6–8]. These latter changes have been posed as consequences of interrupting the natural traction provided by hyolaryngeal movement on upper oesophageal sphincter opening [8–10]. Patient reports of post-laryngectomy dysphagia reflects these physiological deficits with frequent complaints/challenges including effortful bolus transit, post-swallow residue and oral or nasal regurgitation necessitating mealtime compensation (i.e., diet modification, small regular meals due to duration of intake) [3, 5, 11, 12]. These and other swallowing issues often make it difficult for individuals to participate in family gatherings and social engagements which has associated negative consequences for psychological wellbeing and quality of life (QoL) [3, 4, 13–15].

The evaluation of dysphagia in the general (non-laryngectomy) population commonly begins with a clinical assessment undertaken by the speech pathologist. The standard clinical swallow examination (CSE) is a fundamental component of dysphagia assessment used across patient populations [16–18]. The overall purpose of this process is to identify key swallow symptoms, most significantly, external indicators of potential airway invasion (e.g., voice change, throat clearing post swallow) and difficulties with bolus clearance (e.g., multiple swallows, sensation of pharyngeal residue). From these observations, clinicians can infer potential underlying swallow pathophysiology in order to guide management decisions including instrumental assessment referral [17]. This is a dynamic process that begins with collating medical background and patient-reported swallow history to build a clinical hypothesis [19–21]. In the standard CSE, assessment of cranial nerve function is then conducted with one of the more critical observations being the assessment of voice quality and cough strength to expose potential disturbance of airway protection. Various food and fluid consistencies are then trialed to evaluate the efficiency and completeness of bolus transit as well as to expose signs of potential airway invasion [22].

Given the standard CSE was not designed specifically for the laryngectomy population, the anatomical and physiological changes resulting from laryngectomy surgery renders a number of these CSE components irrelevant. The most obvious difference is the removal of the larynx, and separation of the airway and the oesophagus. Aspiration of food/fluids can no longer occur post laryngectomy, and the assessment of voice quality and cough strength is not required. Removal of the larynx combined with pharyngeal reconstruction (neopharynx) also changes the soft tissue landscape which uniquely impacts the swallowing process. Disturbance of standard pharyngeal valving systems and pressure differentials can result in prolonged, effortful and inefficient bolus transit through the (neo)pharynx and into the oesophagus [3, 5, 11, 12]. Post-operative structural complications such as strictures and anterior pouches (i.e., pseudoepiglottis) can also reduce the ease and efficiency of bolus passage [4, 5, 23, 24]. Together, these structural and functional changes can frequently contribute to reflux and regurgitation (backflow) of food and fluid [1, 25]. As these swallow symptoms and their underlying physiology are fundamentally different from other patient populations, they are not adequately represented or examined using a standard CSE.

Whilst the concept of using a CSE to assess dysphagia is evident within laryngectomy swallowing literature, the assessment components themselves have not been detailed or examined specifically for this population. For example, in the systematic review by Terlingen et al. [3], 44 studies reporting on swallow function post total laryngectomy, were reviewed for diagnosis and treatment of dysphagia. Of the 40 studies reporting on swallow assessment processes, no study described a comprehensive CSE procedure. Instead, the majority used instrumental assessments, patient self-reported questionnaires or clinician reported tools not specifically designed for the laryngectomy population (e.g., SWAL-QoL outcomes tool for oropharyngeal dysphagia in adults, therapy outcome measure (TOMS)—dysphagia scale) or a combination of the two. Five of the reviewed studies conducted semi-structured interviews with patients [26–30] to gather information on swallowing function and outcomes. Whilst interview detail varied, all five studies documented the diet texture tolerated by the patient. One study incorporated items gathering patient perceived swallowing disability, handicap and wellbeing/distress (TOMS—dysphagia scale) [29], while another study, reported undertaking a more extensive (23 item) interview that asked patients about laryngectomy-specific swallow symptoms, however, details of the included questions were not provided in full [27]. Terlingen et al. [3] described that “clinical assessment” was reported in 5 of the examined studies, however, on closer review, assessment was again limited to the diet texture tolerated [26, 28, 30–32]. One of the 44 studies described using the 100 ml water swallow test to assess swallow capacity (millilitres swallowed divided by time taken in seconds); this was the only reported clinical measure [33]. In a systematic review by Mahalingam et al. [4], this time examining swallowing outcome measures post pharyngolaryngectomy, instrumental assessment and patient-reported outcomes were the most commonly applied tools. Again, 12 of the 15 studies recorded diet texture tolerated, and no information was provided on the process of any clinical assessment undertaken [4].

Given that the typical components of standard (non-laryngectomy) CSE tools are not sensitive to laryngectomy swallow features, and the current literature fails to adequately describe a comprehensive and consistent swallow assessment process for this population, formal exploration of core assessment elements is required. The present study is part of a research program that seeks to gain agreement on relevant tasks, measures and observations considered essential to clinical (CSE) and instrumental evaluation (videofluoroscopic swallow study, VFSS) of laryngectomy swallowing function. Given the utility and importance of VFSS in post laryngectomy swallow management, the core elements of radiological swallow evaluation for this population have been examined in a separate e-Delphi process. The aim of the current study was to use an expert consensus building process to develop a draft laryngectomy CSE framework that could be used to support the assessment of laryngectomy dysphagia in clinical practice and research.

## Methods

### Research Design

A four-round international e-Delphi process was undertaken between August 2019 and August 2022. The survey process was paused during peak periods of the COVID-19 pandemic globally due to participants’ fluctuating workplace demands. Ethical approval was obtained from The University of Queensland’s Health and Behavioural and Sciences Human Research Ethics Committee (#2018002414).

### The Delphi Technique

An online version of the traditional Delphi consensus building process was used in this study [34]. This iterative process uses successive questionnaire rounds to establish expert consensus on a particular issue or topic [35, 36]. The use of online questionnaires have become popular due to their capacity to facilitate efficient international consensus research [34, 37], hence supporting the context of this study. The Delphi, and modified online ‘e-Delphi’ technique, are increasingly evident in health research to establish various clinical resources and outcomes (e.g., practice guidelines, assessment measures, research indictors and clinical priorities) [38, 39]. Specific to dysphagia, Delphi processes have been used effectively to achieve consensus on a number of different purposes including the development of a swallow risk screening tool and best practice guidelines in cervical spinal injury [40] and the objectives of dysphagia education intervention for patients and caregivers [41]. Within the field of head and neck cancer, Delphi processes have produced consensus statements for patient management guidelines (e.g., dysphagia risk factors, screening and surveillance) [42].

### Participants and Recruitment

Speech pathologists were recruited for this study using purposeful sampling. An invitation to participate was distributed to individual clinicians with known experience in laryngectomy management as well as to subscribers of relevant national and international special interest groups and clinical excellence networks (i.e., laryngectomy, head and neck cancer). This allowed for recruitment of speech pathologists from a diverse sample of countries and settings (e.g., metropolitan and regional hospitals, private and public centres). Speech pathologists were eligible to participate if they had completed 8–10 CSEs with individuals post laryngectomy in the preceding 5 years. Once consented, all participants completed a demographics survey to confirm eligibility. No additional exclusion criteria were set. All participants provided informed, written consent prior to participation and anonymity was maintained to limit potential group conformity.

### Data Collection and Analysis

A series of surveys were developed for data collection. This incorporated an initial demographics survey followed by four rounds of e-Delphi surveys. All surveys were accessed via the online platform, SurveyMonkey (www.surveymonkey.com).

#### Demographic Survey

This preliminary questionnaire requested participants to record information about their country of residence, clinical work setting and facility location (e.g., metropolitan/regional), whether their facility performs laryngectomy/pharyngolaryngectomy surgeries and if so, the number performed annually. They were also asked to report their years of clinical practice and their experience in laryngectomy dysphagia management including if they currently conducted CSEs and the number they had completed in the last 5 years. Finally, they were asked if they had previously undertaken or participated in laryngectomy research.

#### e-Delphi Survey Development

Traditional Delphi methodology uses the first round of the process to brainstorm and generate ideas on a specific topic or issue [35, 43]. Due to high variability in the measures used across laryngectomy swallow assessment literature, this e-Delphi process adopted the only validated, laryngectomy specific patient-reported measure as the stimulus for the Round 1 survey. The swallowing outcome after laryngectomy (SOAL) [44] asks patients to rate how frequently they experience 17 dysphagia related symptoms and associated limitations (i.e., no, a little, a lot and if a lot, then does this bother you?) [44, 45]. Developed by two focus groups consisting of speech pathologists and individuals post laryngectomy, it is a screening tool designed to identify specific areas of swallowing concern and/or monitor changes over time for clinical management and/or laryngectomy research. Preliminary validation of the SOAL found the assessment items had positive and significant correlation with the presence of dysphagia as identified on VFSS [45]. Questions 2–12 of the SOAL ask the patient to grade 11 specific dysphagia symptoms (e.g., need for multiple swallows to get food/fluids down, frequency of food/fluids regurgitation into the mouth or nose). These 11 items were grouped into three categories (fluids, foods and other symptoms or required mealtime strategies) to form the stimulus questions for Round 1 of the e-Delphi process (Table 1). The remaining SOAL items (i.e., Questions 1, 13–17) were not included as these questions are not explicitly related to the swallowing process.

**Table 1:** Swallowing outcomes after laryngectomy (SOAL) stimulus items

SOAL question
Fluids
Do you have a problem swallowing thin liquids (tea, water, juice)? *Unrelated to voice prosthesis leaking*
Do you have a problem swallowing thick liquids (soup, milkshake, supplement drinks)?
Do liquids stick in your throat when you swallow?
Do you need to swallow many times on each mouthful to help the (food or) drink go down?
Foods
Do you have a problem swallowing soft/mashed foods (macaroni cheese, shepherd’s pie)?
Do you have a problem swallowing dry solid food (bread, biscuits)?
Does food stick in your throat when you swallow?
Do you need to swallow liquid to help the food go down?
Do you need to swallow many times on each mouthful to help the food (or drink) go down?
Other symptoms or required mealtime strategies
Does food or liquid come back up into your mouth or nose when you eat or drink?
Do you avoid certain foods because you cannot swallow them?
Does it take you longer to eat a meal?

### Round One: Item Generation

**Fig. 1:**
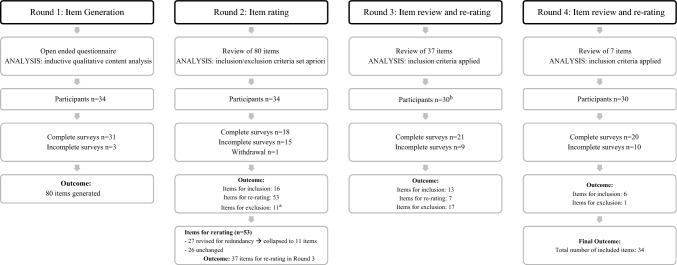
Summary of e-Delphi process and results.^a^Exclusion criteria not met and therefore adjusted. ^b^Participants who did not complete Rounds 1 and 2 were not contacted in Round 3 (n = 3), participant withdrawal (n = 1)

A summary of the e-Delphi process undertaken for this study is outlined in Fig. 1. In the first round, items 2–12 of the SOAL were presented as stimulus questions in the online survey. Preceding each SOAL question, participants were asked “Based on your clinical experience, what would you consider are the best methods to clinically assess each of the following patient-reported symptoms?”. Responses were requested in two parts; (1) clarifying questions that would be asked to the patient and (2) a description of the tasks, measures and observations that they would use to assess each symptom. Prior to commencing the survey an example question and response based on a standard CSE was provided. In this example, tasks were described as the action or activity performed by the patient (e.g., complete food/fluid trials, trial a compensatory manoeuvre) with measures and observations described as objective quantifications (e.g., number of swallows) and subjective detections (signs of discomfort and effort), respectively. In addition, participants were asked to describe any oromotor assessment tasks they typically undertook during a laryngectomy CSE and any other tasks, measures and observations conducted that were not prompted by the SOAL questions.

Data Analysis: Inductive qualitative content analysis as outlined by Graneheim and Lundman [46] was applied to Round 1 responses. All participant responses were reviewed for meaning units and subsequently filtered into codes, subcategories, categories and themes. Survey responses were initially reviewed several times by the principal investigator (SW) to facilitate immersion in the data as well as an overview of the content [47]. All raw data responses were reviewed by the two co-investigators (BB and CB) and extraction of meaning units was completed as a research team to gain consensus. Approximately 70% of meaning unit categorization into codes, categories, subcategories, and themes was conducted collaboratively by the three investigators. The remaining analysis and categorization of meaning units was undertaken by the principal investigator with the final analysis reviewed in full by the co-investigators for agreement.

### Round Two

All participants who engaged in Round 1 were invited to participate in Round 2. A summary of the Round 1 results was provided to the participants for review and rating in Round 2. The survey format for Round 2 followed the content analysis structure: overarching theme (e.g., patient interview), category (e.g., temporal aspects of swallowing) and corresponding subcategories (e.g., “How long does it take you to finish a meal?”). Each subcategory formed a survey “item” for rating. Participants were asked to rate each proposed assessment item (i.e., subcategory) for importance of inclusion in a laryngectomy CSE using the Grading of Recommendations Assessment, Development and Evaluation (GRADE) working group rating scale [48]. The scale utilizes a 1–9 range that divides scoring into three categories: limited importance (rating 1–3), important but not critical (rating 4–6) and of critical importance (rating 7–9).

Data analysis: results were analysed using descriptive statistics (mean, median, range, minimum, maximum) as well as GRADE scale categories. Each item was reviewed for the number and percentage of participants rating it as: limited importance (rating 1–3), important but not critical (rating 4–6) and critical (rating 7–9). Consensus levels were defined prior to study commencement in line with previous e-Delphi studies [49, 50]. Inclusion was agreed at ≥ 75% of panel members scoring an item of critical importance (rating 7–9) and ≤ 15% of panel members rating an item of limited importance (rating 1–3) for inclusion in a laryngectomy CSE. Exclusion was set at ≥ 75% of the group scoring an item of limited importance (1–3) and ≤ 15% of the panel rating an item as “critically important” (7–9). Items falling outside these criteria were considered ‘inconclusive’ and formed the basis of Round 3. No minimum panel response rate was set due to the small number of participants.

### Round Three

At the commencement of the Round 3 survey, Round 2 results were presented for participants to view. Summary statistics of ratings were provided for each subcategory (i.e., mean, median, percentage rating 7–9 “of critical importance”) including which items reached consensus and those that were excluded. The inconclusive items were then presented under the same category and theme headings as per the Round 2 survey for re-rating by participants. Re-rating occurred using the same GRADE 9-point scale. Round 2 frequency data was not presented to participants in the body of the survey (i.e., Round 2 summary data presented alongside each item as it was re-rated) to avoid panel bias [51]. Participants who did not complete Rounds 1 and 2 were not contacted to participate in Round 3.

Data analysis: data was analysed as per Round 2 analysis. Inclusion and exclusion criteria remained unchanged.

### Round Four

The Round 4 survey included Round 3 results in the form of summary statistics as per previous rounds. Re-rating of the inconclusive items followed the same procedure as for Rounds 2 and 3. Participants who completed either Rounds 1 and 2 or Rounds 1 and 3 were invited to complete Round 4.

Data analysis: results were analysed as per Rounds 2 and 3.

## Results

### Participants

An overview of participant involvement across the 4-round e-Delphi process is outlined in Fig. 1. A total of 34 speech pathologists comprising of Australian (n = 18, 53%) and international (n = 16, 47%) clinicians participated (Table 2). The majority of clinicians were working in public hospital facilities (n = 29, 85%) based in metropolitan locations (n = 25, 74%) that conducted laryngectomy surgeries onsite (n = 29, 85%). The number of surgeries performed annually varied across sites (range < 10–30 per annum). Nearly half of the participants (n = 15, 44%) had been working clinically for over 20 years, and greater than 50% reported 11–20 years of practice in laryngectomy dysphagia management (n = 21, 62%). Most conducted laryngectomy CSEs as part of their clinical position (n = 30, 88%) and almost half of the cohort (n = 15, 44%) reporting they had conducted > 41 CSEs in the last 5 years. Two thirds of participants (n = 22, 65%) reported undertaking or participating in research related to laryngectomy.

**Table 2:** Participant demographics

Participant characteristics	Number of participants (%)
Country	
Australia	18 (53)
United Kingdom	9 (26)
USA	4 (12)
Canada	1 (3)
New Zealand	1 (3)
Sweden	1 (3)
Clinical settings^a^	
Public hospital	29 (85)
Private hospital	4 (12)
Private practice	1 (3)
Other *(i.e., Academic hospital/clinic, Cancer Centre)*	2 (6)
Facility location	
Metropolitan	25 (74)
Regional	9 (26)
Facility performs laryngectomy/pharyngolaryngectomy surgeries^b^	
Yes	29 (85)
No	4 (12)
Approximate number of surgeries per year^b^	
< 10	12 (35)
10–19	7 (21)
20–30	8 (24)
> 30	1 (3)
Years of clinical practice experience	
0–10	7 (21)
11–20	12 (35)
21 +	15 (44)
Years of experience in dysphagia management following laryngectomy	
0–10	8 (24)
11–20	21 (62)
21 +	5 (15)
Number of CSE following laryngectomy in the last 5 years	
0–10	3 (9)
11–20	4 (12)
21–40	12 (35)
41 +	15 (44)
Current position conducts CSEs following laryngectomy	
Yes	30 (88)
No	4 (12)
Undertaken or participated in laryngectomy research	
Yes	22 (65)
No	12 (35)

^a^participants could select > 1 option, ^b^one incomplete response

### Round 1

The Round 1 survey was completed by 31 participants (3 incomplete surveys) (Fig. 1). Four themes emerged from the data; *1. General function and medical condition, 2. Patient interview, 3. General oropharyngeal tasks and observations, 4. Swallow-specific tasks, measures and observations* (Table 3). Themes 1 and 2 were generated from clarifying questions that the speech pathologists stated they would ask the patient based on the SOAL stimulus items. Themes 3 and 4 were generated from the tasks, measures and observations that the clinicians stated they would use to investigate the swallow symptoms listed in the 11 SOAL items. A total of 25 categories and 80 subcategories were identified across the four themes with a greater number of these generated for Theme 2—Patient interview (n = 39 statements) and Theme 4—Swallow-specific tasks, measures and observations (n = 21 statements) (Supplementary Table 1). Each subcategory reflected a potential item to be included in a laryngectomy CSE.

**Table 3:** Summary of content analysis from Round 1 responses

Theme	Category
Section one—patient history and interview	
1. General functioning and medical condition	1. Medical history 2. Weight loss and dehydration 3. Pain and swelling 4. Psychosocial: QOL, eating in public, distress/anxiety, self-care
2. Patient interview	5. Temporal aspects of swallowing 6. Consistencies of oral intake 7. Bolus direction and flow 8. Strategies to optimise oral intake
Section two—clinical swallow assessment—non swallow	
3. General oropharyngeal tasks and observations	9. Cranial nerve assessment 10. Oropharyngeal condition
Section three—clinical swallow assessment—swallow	
4. Swallow tasks, measures and observations	11. Tasks 12. Measures 13. Observations 14. Patient report during clinical assessment 15. Onward referrals/investigations

*QOL* quality of life

### Round 2

Round 2 was completed by 18 participants (15 incomplete surveys, 1 withdrawal; Fig. 1). Participants were presented with 80 items emerging from the Round 1 analysis for rating in Round 2 (Table 4). Sixteen items met the criteria for inclusion, however, no items met the exclusion criteria. Given that a component of consensus building includes elimination of low priority or non-consensus items [52], in the occurrence of a high number of these, the research team redefined the exclusion criteria to facilitate this step. Eleven of the non-consensus items (n = 64) were rated of critical importance by ≤ 33% of participants (Table 4). As these items were unlikely to reach consensus for inclusion in subsequent rounds, the exclusion criteria was reset at this level. In addition, the remaining non-consensus items (n = 53) were reviewed by the research team for construct similarity and redundancy to minimise any duplication as well as reduce the Round 3 survey length for participants. Repetition was eliminated where the same question or task was repeated for both food and fluids (Table 5). Items with similar concepts were also combined where appropriate. Twenty-seven items were revised and condensed into 11 items. Hence, non-consensus items were reduced from 53 to 37 items, which were then rated by the participants in the Round 3 survey.

**Table 4:** Round 2 rating results

Clinical assessment items: questions, tasks, measures and observations		Rated of Critical Importance by	Min	Max	Median	**Mean**
Include	Confirm any previous surgical and non-surgical treatment for dysphagia (e.g. dilatation, botox, dysphagia rehabilitation exercises etc.) and outcomes of this treatment	100%	7	9	9	8.6
	Discuss outcome with ENT / head and neck team	100%	7	9	9	8.5
	Confirm patient’s surgical treatment history (e.g., recency, primary vs salvage, method of repair)	94%	5	9	9	8.3
	Confirm patient’s non-surgical treatment history	89%	5	9	8	7.9
	Confirm any previous assessment for dysphagia	89%	5	9	8	7.9
	What is your current diet?	89%	6	9	8	8.0
	Assess CN XII function	89%	5	9	8	7.7
	Consider referral for VFSS	89%	6	9	8.5	8.1
	Have you lost any weight?	78%	4	9	8.5	7.7
	Do you experience difficulties with particular food consistencies? If so, please describe	78%	5	9	8	7.6
	Which foods are easier and harder to swallow?	78%	5	9	7.5	7.6
	What foods do you avoid?	78%	5	9	7	7.4
	Have you noticed a change in your tracheoesophageal voice recently?	78%	5	9	7	7.4
	Have you noticed a change in your voice prosthesis function (e.g., leakage when eating/drinking, shortly after intake)?	78%	3	9	9	7.8
	Examine oral and lingual mucosa	78%	3	9	7.5	7.1
	Evidence of backflow into or out of the oral or nasal cavities	78%	5	9	7	7.3
Review and re-rate	Have you experienced any pain or discomfort on swallowing?	72%	6	9	8	7.6
	Do you have any swelling in the mouth, throat or neck?	72%	5	9	7	7.2
	Does your swallowing impact your QOL?	72%	5	9	8	7.6
	How long has this been happening?	72%	5	9	7	7.3
	Do you think it is getting better or worse over time?	72%	5	9	7	7.4
	Assess CN VII function	72%	4	9	7	7.1
	Assess CN IX function	72%	4	8	7	6.9
	Observe saliva presence and consistency	72%	4	9	7	6.8
	Observe for pressure/effort/struggle on swallowing	72%	5	9	8	7.4
	Observe for quality and tonicity of tracheoesophageal voice	72%	3	9	7	6.9
	How distressing is your swallowing for you?	67%	5	9	7.5	7.5
	Was it a gradual or sudden onset?	67%	6	9	7.5	7.4
	Does it happen every time you eat/drink? (If not, how often?)	67%	3	9	7	7.1
	Do you experience difficulties with particular liquids? If so, please describe	67%	3	9	7	7.1
	Do you suffer from reflux?	67%	3	9	7.5	7.1
	Do you take any medications for reflux? If so, do they help?	67%	3	9	8	7.3
	Examine state of dentition	67%	4	9	7	7.1
	Does this stop you from eating out/impact on your social opportunities?	61%	5	9	7	7.1
	Do you experience nasal regurgitation/backflow? And if so, when does it happen? (e.g., immediately, after a period of time, when you bend over)	61%	2	9	7	6.8
	Assess CN X function (excluding laryngeal tasks)	61%	3	9	7	6.8
	Undertake solid trials using a variety of textures	61%	5	9	7	7.3
	Trial of patient initiated and/or clinically indicated strategies (e.g., food/fluid modifications, compensatory manoeuvres)	61%	4	9	7	7.2
	Ask patient to rate/report on effectiveness of trialed strategies	61%	4	9	7	7.1
	Have you experienced any signs of dehydration (e.g., dark urine, fatigue, thirst)?	56%	4	9	7	6.8
	Does it get easier/harder over the meal/intake?	56%	3	9	7	6.8
	How many times do you need to swallow to get a mouthful of fluid down?	56%	4	9	7	6.6
	How many times do you need to swallow to get a mouthful of food down?	56%	4	9	7	6.7
	How long does the food/fluid get stuck for?	56%	2	9	7	6.1
	Do drinks come back into/out of your mouth or nose?	56%	4	9	7	6.9
	Does food come back into/out of your mouth or nose?	56%	4	9	7	6.9
	Observe voice quality in conversation pre/post fluid trials (e.g., maximum phonation time, speech sample)	56%	3	9	7	6.9
	Observe oral phase and mastication for difficulty; duration, effort, residue etc	56%	4	9	7	6.7
	Confirm any new/pre-existing physical or medical issues that impact capacity for self-care (e.g., poor exercise tolerance, reduced fine motor skills)	50%	5	9	6.5	6.7
	How long does it take you to drink a cup of liquid?	50%	4	9	6.5	6.7
	How long does it take you to finish a meal?	50%	4	9	6.5	6.8
	Is it better or worse at certain times of the day?	50%	3	9	6.5	6.4
	If food sticks in your throat, can you point to where you feel it is sticking (in your throat)?	50%	2	9	6.5	6.1
	Have you noticed a change in your tracheoesophageal voice during drinking?	50%	2	9	6.5	6.0
	Have you noticed a change in your voice prosthesis fit?	50%	3	9	7	6.8
	Undertake fluid trials including increasing viscosity and altering bolus size	50%	2	9	6.5	6.7
	Count the number of spontaneous swallows to clear each bolus of food and fluid	50%	3	9	6.5	6.7
	Observe natural head and neck position during eating	50%	4	9	6.5	6.2
	Observe natural head and neck position during drinking	50%	4	9	6.5	6.2
	Ask patient to report on the swallowing symptoms during trials as compared to typical function	50%	4	9	6.5	6.7
	Have you noticed a change in your tracheoesophageal voice during eating?	44%	2	9	6	5.9
	Do you use any strategies to help clear food residue in your throat? (e.g. fluid flushes, adding sauces gravies, changes in temperature/carbonation etc.)	44%	3	9	6	6.9
	Do you alter your position/posture to help your swallowing?	44%	4	9	6.5	6.6
	Assess CN V function	44%	4	9	6.5	6.6
	Request the patient complete a food diary for review	39%	2	9	6	5.8
	Do you use any strategies to make swallowing fluid easier? If so, what are these?	39%	5	9	6	6.8
	Do you use any strategies to make swallowing food easier? If so, what are these?	39%	5	9	6	6.9
	Measure voice using maximum phonation time	39%	1	9	6	5.4
	Consider referral for Videomanometry	38%	1	9	5	4.8
Exclude	When drinking, do you take one sip at a time or drink continuously?	33%	2	9	6	6.2
	Do smaller or bigger mouthfuls of food make swallowing easier?	33%	3	9	6	6.3
	Do you use any strategies to help clear fluid residue in your throat? (e.g., extra swallows, eating at the same time, using thinner liquids, straw use etc.)	33%	3	9	6	6.6
	(If you experience xerostomia), what do you do to help this and does it improve your swallowing?	33%	2	9	6	5.9
	Do you experience a dry mouth (xerostomia)? If so, how does this impact fluid moving through your mouth?	28%	4	9	5.5	5.9
	Do you experience a dry mouth (xerostomia)? If so, how does this impact food moving through your mouth?	28%	4	9	6	6.2
	Do smaller or bigger sips of fluid make swallowing easier?	28%	3	9	6	6.2
	Consider referral for FEES	27%	1	9	5	5.2
	Measure time and number of swallows to clear a set volume of fluid (e.g., 100 ml Water Swallow Test)	11%	1	9	5	4.8
	Collect Iowa Oral Performance Instrument (IOPI) measures of lingual strength	6%	1	7	4	3.8
	Undertake timed 100 ml Water Swallow Test	6%	1	7	4.5	4.3

*ENT* ear, nose and throat, *CN* cranial nerve, *VFSS* videofluoroscopic swallowing study, *QOL* quality of life, *FEES* fibreoptic endoscopic evaluation of swallowing

**Table 5:** Round 2 items condensed for re-rating in Round 3

Round 2 items (n = 27)	Collapsed items (round 3) (n = 11)
How long does it take you to drink a cup of liquid? How long does it take you to finish a meal?	How long does it take you to drink a) a cup of liquid and b) finish a meal?
How long has this been happening? Was it a gradual or sudden deterioration/change?	When and how did this start (e.g., gradually or suddenly)?
Does it get easier/harder over the meal/intake? Is it better or worse at certain times of the day?	Is it better or worse a) over the meal/drink or b) at certain times of the day?
How many times do you need to swallow to get a mouthful of fluid down? How many times do you need to swallow to get a mouthful of food down?	How many swallows do you need to get a mouthful of a) fluid or b) food down?
Do drinks come back into/out of your mouth or nose? Does food come back into/out of your mouth or nose? Do you experience nasal regurgitation/backflow? And if so, when does it happen (e.g., immediately, after a period of time, when you bend over)?	Do a) food or b) drinks come back into or out of your nose or mouth? If so, when does this occur (e.g., immediately, after a period of time, when you bend over)?
Have you noticed a change in your tracheoesophageal voice during drinking? Have you noticed a change in your tracheoesophageal voice during eating?	Have you noticed a change in your tracheoesophageal voice during a) eating or b) drinking?
Do you suffer from reflux? Do you take any medications for reflux? If so, do they help?	Do you suffer from reflux? If so, do you take any medications, and do they help?
Do you use any strategies to make swallowing fluid easier? If so, what are these? Do you use any strategies to make swallowing food easier? If so, what are these? Do you use any strategies to help clear food residue in your throat (e.g., fluid flushes, adding sauces gravies, changes in temperature/carbonation etc.)? Do you alter your position/posture to help your swallowing?	Do you use any strategies to make swallowing a) fluid or b) foods easier (e.g., fluid flushes, adding sauces or gravies, changing you position/posture)? If so, what are these?
Assess CN V function Assess CN VII function Assess CN IX function Assess CN X function (excluding laryngeal tasks)	Assess CN V, VII, IX & X function
Undertake fluid trials including increasing viscosity and altering bolus size Undertake solid trials using a variety of textures	Undertake a) fluid and b) solids trials using increasing viscosity and texture respectively
Observe natural head and neck position during eating Observe natural head and neck position during drinking	Observe head and neck position during oral trials

*CN* cranial nerve

### Round 3

The third e-Delphi round was completed by 21 participants (Fig. 1). A further 13 items reached consensus for inclusion (Table 6). Seven items were on the cusp of inclusion and progressed to review in a fourth round. The remaining items (n = 17) did not meet the revised exclusion criteria and had failed to meet inclusion criteria, and so did not progress.

**Table 6:** Round 3 rating results

Clinical assessment items: questions, tasks, measures and observations		Rated of Critical Importance by	Min	Max	Median	Mean
Include	Do you think it is getting better or worse over time?	100%	7	9	8	8.1
	When and how did this start (i.e., gradually or suddenly)?	100%	7	9	8	8.1
	Does it happen every time you eat/drink? (If not, how often?)	90%	4	9	8	7.7
	Does your swallowing impact your QOL?	86%	4	9	8	7.6
	Do a) drinks or b) food come back into or out of your nose or mouth? If so, when does this occur? (e.g., immediately, after a period of time, when you bend over)?	86%	5	9	8	7.6
	Do you suffer from reflux? If so, do you take any medications, and do they help?	81%	4	9	8	7.7
	Observe for pressure/effort/struggle on swallowing	81%	5	9	8	7.6
	How many times do you need to swallow to get a mouthful of a) fluid and/or b) food down?	81%	5	9	7	7.4
	Have you experienced any pain or discomfort on swallowing?	81%	3	9	8	7.7
	Do you use any strategies to make swallowing a) fluid and/or b) foods easier (e.g., fluid flushes, adding sauces or gravies, changing your position/posture)? If so, what are these?	81%	5	9	8	7.6
	Does this stop you from eating out/impact on your social opportunities?	81%	4	9	7	7.2
	How distressing is your swallowing for you?	81%	4	9	8	7.4
	Ask patient to rate/report on effectiveness of trialled strategies	76%	5	9	8	7.7
Re-rate	Undertake a) fluid and/or b) solids trials using increasing viscosity and texture respectively	71%	3	9	8	7.3
	Observe oral phase and mastication for difficulty; duration, effort, residue etc	71%	2	9	8	7.2
	Ask patient to report on the swallowing symptoms during trials as compared to typical function	71%	5	9	7	7.2
	Have you noticed a change in your tracheoesophageal voice during a) drinking and/or b) eating?	71%	2	9	8	6.9
	Do you have any swelling in the mouth, throat or neck?	71%	3	9	7	6.9
	How long does it take you to a) drink a cup of liquid and/or b) finish a meal?	71%	4	9	8	7.3
	Trial of patient initiated and/or clinically indicated strategies (e.g., food/fluid modifications, compensatory manoeuvres)	71%	4	9	8	7.4
Exclude	Observe for quality and tonicity of tracheoesophageal voice	67%	2	9	7	6.8
	Do you experience difficulties with particular liquids? If so, please describe	67%	2	9	7	6.8
	Is it better or worse a) over the drink/meal or b) at certain times of the day?	67%	3	9	7	6.6
	Request the patient complete a food diary for review	62%	1	9	6	5.9
	Have you noticed a change in your voice prosthesis fit?	62%	3	9	7	6.9
	Count the number of spontaneous swallows to clear each bolus of food and fluid	62%	5	9	7	7.1
	Observe voice quality in conversation pre/post fluid/food trials (e.g., maximum phonation time, speech sample)	57%	2	9	7	7.0
	Assess CN V, VII, IX & X (relevant tasks) function	57%	3	9	7	6.5
	Confirm any new/pre-existing physical or medical issues that impact capacity for self-care (e.g., poor exercise tolerance, reduced fine motor skills)	57%	1	9	7	6.4
	If food sticks in your throat, can you point to where you feel it is sticking (in your throat)?	57%	2	9	7	6.5
	Examine state of dentition	57%	3	9	7	6.5
	Observe head and neck position during oral trials	52%	4	9	7	6.6
	How long does the food/fluid get stuck for?	48%	3	9	6	6.3
	Have you experienced any signs of dehydration (e.g., dark urine, fatigue, thirst)?	38%	1	8	6	5.6
	Observe saliva presence and consistency	38%	3	9	6	6.2
	Consider referral for Videomanometry	33%	2	9	6	5.7
	Measure voice using maximum phonation time	33%	2	9	6	5.3

*QOL* quality of life

### Round 4

Twenty participants completed the fourth round. Six of the seven items met criteria for inclusion (Table 7), resulting in a total of 34 items reaching consensus for inclusion in a laryngectomy CSE (Table 8).

**Table 7:** Round 4 rating results

Clinical assessment items: questions, tasks, measures and observations		Rated of Critical Importance by	Min	Max	Median	Mean
Include	Ask patient to report on the swallowing symptoms during trials as compared to typical function	85%	4	9	8.5	7.8
	How long does it take you to a) drink a cup of liquid and/or b) finish a meal?	84%	6	9	8	7.7
	Undertake a) fluid and/or b) solids trials using increasing viscosity and texture respectively (as indicated)	80%	4	9	8.5	7.9
	Trial of patient initiated and/or clinically indicated strategies (e.g., food/fluid modifications, compensatory manoeuvres)	80%	4	9	8	7.6
	Observe oral phase and mastication for difficulty; duration, effort, residue etc	75%	3	9	8	7.2
	Have you noticed a change in your tracheoesophageal voice during a) drinking and/or b) eating?	75%	1	9	7	7.1
	Do you have any swelling in the mouth, throat or neck?	37%	3	9	5.5	5.8

**Table 8:**
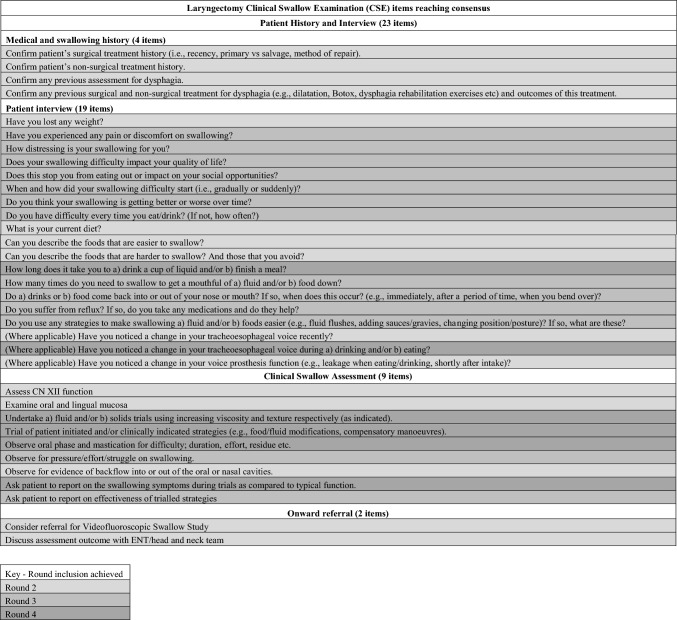
Final consensus results

*CN* cranial nerve, *ENT* ear, nose and throat

### Final Consensus Results

Four overarching themes were evident in the final results: (1) Medical and swallow history; (2) Patient interview; (3) Clinical swallow assessment; and (4) Onward referral. Of the 34 items reaching final consensus, more than two thirds (n = 23) related to information gathering (Theme 1). Twelve of the items focused on the patient’s medical and swallow history and 11 items sought patient report of their swallowing status and dysphagic features. In comparison, 11 of the 34 consensus items pertained to the observations during the clinical assessment itself; with 7 of these items constituting specific swallowing tasks and observations and four items relating to patient feedback during the oral trial. Theme 4 (Onward referral) included just two items specifying consideration of instrumental assessment and onward specialist medical review. The final list of consensus items were sent to participants for their review and comment. Positive feedback was obtained and, as no additional comments or changes were received, the CSE laryngectomy framework was finalised.

## Discussion

This study utilised an e-Delphi process with experienced international speech pathologists to reach consensus on assessment items considered critical to a laryngectomy CSE. The opening round yielded an extensive list of 80 proposed items. This broad starting point was effectively narrowed in subsequent rounds to a more clinically appropriate list of 34 core items. In keeping with the standard CSE process, the final consensus framework comprised of items incorporating medical history and patient interview as well as clinician directed tasks and observations. Importantly, the core items examine key symptoms commonly observed in laryngectomy dysphagia and explore patient-reported changes and experiences associated with their swallow function. Therefore, this initial framework can be considered as a first step in the development of a comprehensive CSE for use with the laryngectomy population in both clinical practice and research.

In keeping with a standard CSE, patient history and interview (Theme 1) was the most extensive section of the final consensus framework. While ‘patient interview’ has been referred to in the laryngectomy literature, information on the questions used has been limited or incomplete [3, 27, 53]. The current study has contributed important new information; a detailed list of laryngectomy-specific questions exploring not only dysphagic features but also its impact on swallowing status, mealtime participation and quality of life. A potential reason for the high number of information gathering items may be attributable to the e-Delphi design. The survey stimulus items were derived from a laryngectomy-specific, patient-reported tool (the SOAL) and presented as patient-reported symptoms. In addition, participants were explicitly instructed to provide “clarifying questions that you would ask the patient” in response to each swallow symptom. Hence, the decision to use relevant SOAL questions likely facilitated participants to generate a comprehensive list of symptoms and patient-experience items, rather than primarily focusing on clinician-directed components to examine swallowing function.

Importantly, whilst the SOAL stimulus items were foreseeably reflected in the final CSE consensus items, duplication was minimal. Instead, both tools provide complementary information when assessing laryngectomy swallow function. The patient-reported SOAL confirms whether a patient experiences a particular swallow symptom and with what frequency it occurs (e.g., “Do food or drink come back into nose or mouth when you eat or drink?”—no, a little or a lot), whereas the CSE explores the nature and timing of the difficulty (e.g., “If so, when does this occur? E.g., immediately, after a period of time, when you bend over?”) and the attempts to stimulate its occurrence during food/fluid trials. These tools could be used separately or, more ideally, in combination, with the SOAL completed prior to the appointment to guide the speech pathologist on specific lines of questioning during the patient interview and areas to target during the clinical swallow assessment.

Participants proposed a comprehensive list of swallow tasks, measures and observations in Round 1 and rapidly refined this list to 7 targeted items. Assessment of the hypoglossal nerve (CNXII) was the only oromotor component to reach inclusion. Whilst the hypoglossal nerve is not typically damaged, it is described as being vulnerable during the laryngectomy procedure with a reported incidence of 5% of patients experiencing iatrogenic palsy [54, 55]. Hence participants agreed screening of CNXII function was essential to identify potential nerve injury and its impact on swallowing function. The consensus items also included tasks and observations common to the standard CSE (e.g., oral cavity review, food and fluid trials) [16, 18] and laryngectomy-specific swallow symptoms. For example, early consensus was reached on items such as “observe for pressure/effort/struggle on swallow” and “observe for evidence of backflow via oral/nasal cavities”, both of which infer potential anatomical complications and/or physiological breakdowns confirmed in the laryngectomy literature [5, 11, 12]. Finally, onward referral for a VFSS was also prioritised early by participants, confirming the panel’s belief that a CSE does not negate the need to directly visualise the swallowing process to diagnose deficits. This outcome was anticipated due to the wide range and variability of anatomical, motor and sensory changes post-laryngectomy, and predominance for VFSS use in the laryngectomy literature.

Three questions relating to tracheoesophageal voicing and its relationship with eating and drinking reached the inclusion criteria. This outcome occurred in the absence of stimulus (SOAL) questions relating to voicing. Tracheoesophageal voicing, the gold standard in laryngectomy communication rehabilitation, is achieved via a silicon voice prosthesis positioned within the muscular wall between the trachea and oesophagus (surgical voice restoration). Voice prothesis functioning and tracheoesophageal voice quality can be affected by laryngectomy swallow mechanics [56, 57]. Participants prioritised assessment of these features to provide insight into potential swallowing deficits. For example, persistent central leakage through a voice prosthesis may be caused by excessive negative oesophageal pressure during swallowing [58, 59]. Whilst not all individuals undergo surgical voice restoration, inclusion of these items in a laryngectomy CSE could be useful in highlighting potential swallowing issues for a high proportion of the population who use a voice prosthesis. Three swallow-related wellbeing items (i.e., distress, QoL and social limitation) reached consensus in Rounds 2 and 3 of the process. As discussed above, one reason for this could be due to the SOAL stimulus questions, which requested participants to consider patients’ swallowing symptoms and mealtime experiences when generating e-Delphi items. Another reason could be due to increasing reports in the laryngectomy literature of dysphagia related burden, activity limitation and participation restrictions stemming from swallowing and mealtime difficulties (e.g., reduced mealtime enjoyment and engagement, reduced social or communal eating). Similarly reduced quality of life (swallow and health related) and affective symptoms (anxiety, depression, stress, distress) are experienced by approximately one third of the laryngectomy population [15, 60, 61]. The prioritisation of wellbeing items within a laryngectomy CSE not only acknowledges this literature but also international Head and Neck Cancer guidelines which promote consideration of patients’ current and anticipated physical and emotional needs [62, 63]. More specifically, these guidelines task speech pathologists with exploring swallow related wellbeing and facilitating appropriate multidisciplinary intervention [15, 63]. Hence, inclusion of psychosocial items within the laryngectomy CSE prioritises management of these challenges via early identification and onward referral.

Limitations are acknowledged in this study. Participant engagement fluctuated throughout the e-Delphi process. Whilst attrition was anticipated based on reports in previous Delphi literature [34, 64], the extended interruptions between survey rounds due to COVID-19 likely contributed to the one third reduction in completed survey responses for Rounds 2 to 4. Participant fatigue due to the high number of responses required to be entered in Round 1 and rated in Round 2 may have also contributed to this attrition. Although a broad range of speech pathologists participated in the process, it is acknowledged that the participant panel did not represent all countries or ethnic/cultural, socio-economic or healthcare contexts, therefore results may need to be modified/expanded to accommodate local patient needs and healthcare practices in future studies.

## Conclusions

This e-Delphi process is the first study to have explored consensus on the essential components when conducting a CSE to investigate dysphagia following laryngectomy. A panel of international speech pathologists agreed on 34 items incorporating history gathering, patient report and clinical assessment, that examine a broad range of laryngectomy-specific swallowing issues consistent with symptoms raised in the laryngectomy literature. It is hoped that this framework will provide the basis for a more consistent approach when conducting clinical examination of swallowing function post laryngectomy.

## Supplementary Information

Below is the link to the electronic supplementary material.

Supplementary file1 (DOCX 25 kb)

## Data Availability

The data that supports the findings of this study is available from the corresponding author upon request. Data is located in the University of Queensland’s Research Data Manager.
