# Supplementary material for: Clinical Swallow Examination Following Laryngectomy: An International e-Delphi Consensus Process
Source: Dysphagia. 2025 Jan 21;40(4):869–85. doi: 10.1007/s00455-024-10785-0 (PMC12328528; doi:10.1007/s00455-024-10785-0)
Supplement: Supplementary file 1 — Supplementary file1 (DOCX 25 kb) [file 455_2024_10785_MOESM1_ESM.docx]

**Clinical swallow examination following laryngectomy: An international e-Delphi consensus process.**

**Supplementary Table 1**

*Round 1 content analysis themes and subcategories*

| **Theme 1: General functioning and medical condition (n=12)** |
| --- |
| Confirm patient’s surgical treatment history (i.e., recency, primary vs salvage, method of repair). |
| Confirm patient’s non-surgical treatment history. |
| Confirm any previous assessment for dysphagia. |
| Confirm any previous surgical and non-surgical treatment for dysphagia (e.g. dilatation, botox, dysphagia rehabilitation exercises etc) and outcomes of this treatment. |
| Confirm any new/pre-existing physical or medical issues that impact capacity for self-care (i.e., poor exercise tolerance, reduced fine motor skills). |
| Have you lost any weight? |
| Have you experienced any signs of dehydration (e.g., dark urine, fatigue, thirst)? |
| Have you experienced any pain or discomfort on swallowing? |
| Do you have any swelling in the mouth, throat or neck? |
| How distressing is your swallowing for you? |
| Does your swallowing impact your QOL? |
| Does this stop you from eating out/impact on your social opportunities? |
| **Theme 2: Patient interview (n=39)** |
| How long does it take you to drink a cup of liquid? |
| How long does it take you to finish a meal? |
| How long has this been happening? |
| Was it a gradual or sudden onset? |
| Do you think it is getting better or worse over time? |
| Is it better or worse at certain times of the day? |
| Does it happen every time you eat/drink? (If not, how often?) |
| Does it get easier/harder over the meal/intake? |
| What is your current diet? |
| Do you experience difficulties with particular liquids? If so, please describe. |
| Do you experience difficulties with particular food consistencies? If so, please describe. |
| Which foods are easier and harder to swallow? |
| What foods do you avoid? |
| Request the patient complete a food diary for review. |
| Do you experience a dry mouth (xerostomia)? If so,- how does this impact fluid moving through your mouth? |
| Do you experience a dry mouth (xerostomia)? If so,- how does this impact food moving through your mouth? |
| How many times do you need to swallow to get a mouthful of fluid down? |
| How many times do you need to swallow to get a mouthful of food down? |
| If food sticks in your throat, can you point to where you feel it is sticking (in your throat)? |
| How long does the food/fluid get stuck for? |
| Do drinks come back into/out of your mouth or nose? |
| Does food come back into/out of your mouth or nose? |
| Do you experience nasal regurgitation/backflow? And if so, when does it happen? (i.e., immediately, after a period of time, when you bend over) |
| Have you noticed a change in your tracheoesophageal voice recently? |
| Have you noticed a change in your tracheoesophageal voice during drinking? |
| Have you noticed a change in your tracheoesophageal voice during eating? |
| Have you noticed a change in your voice prosthesis fit? |
| Have you noticed a change in your voice prosthesis function (e.g., leakage when eating/drinking, shortly after intake)? |
| Do you suffer from reflux? |
| Do you take any medications for reflux? If so, do they help? |
| Do you use any strategies to make swallowing fluid easier? If so, what are these? |
| Do you use any strategies to make swallowing food easier? If so, what are these? |
| When drinking, do you take one sip at a time or drink continuously? |
| Do smaller or bigger sips of fluid make swallowing easier? |
| Do smaller or bigger mouthfuls of food make swallowing easier? |
| Do you use any strategies to help clear fluid residue in your throat? (e.g., use extra swallows, eating at the same time, using thinner liquids, straw use etc) |
| Do you use any strategies to help clear food residue in your throat? (e.g. fluid flushes, adding sauces gravies, changes in temperature/carbonation etc) |
| Do you alter your position/posture to help your swallowing? |
| (If you experience xerostomia), what do you do to help this and does it improve your swallowing? |
| **Theme 3: General oropharyngeal tasks and observations (n=8)** |
| Assess CN V function |
| Assess CN VII function |
| Assess CN IX function |
| Assess CN X function (excluding laryngeal tasks) |
| Assess CN XII function |
| Examine oral and lingual mucosa |
| Observe saliva presence and consistency |
| Examine state of dentition |
| **Theme 4: Swallow tasks, measures and observations (n=21)** |
| Collect Iowa Oral Performance Instrument (IOPI) measures of lingual strength |
| Undertake fluid trials including increasing viscosity and altering bolus size |
| Undertake timed 100ml Water Swallow Test |
| Undertake solid trials using a variety of textures |
| Trial of patient initiated and/or clinically indicated strategies (i.e., food/fluid modifications, compensatory manoeuvres) |
| Observe voice quality in conversation pre/post fluid trials (i.e., maximum phonation time, speech sample) |
| Count the number of spontaneous swallows to clear each bolus of food and fluid |
| Measure time and number of swallows to clear a set volume of fluid (e.g., 100ml Water Swallow Test) |
| Measure voice using maximum phonation time |
| Observe natural head and neck position during eating. |
| Observe natural head and neck position during drinking. |
| Observe oral phase and mastication for difficulty; duration, effort, residue etc. |
| Observe for pressure/effort/struggle on swallowing. |
| Evidence of backflow into or out of the oral or nasal cavities. |
| Observe for quality and tonicity of tracheoesophageal voice. |
| Ask patient to report on the swallowing symptoms during trials as compared to typical function |
| Ask patient to rate/report on effectiveness of trialed strategies |
| Consider referral for VFSS |
| Consider referral for FEES |
| Consider referral for Videomanometry |
| Discuss outcome with ENT / head and neck team |
